# BAK-Mediated Pyroptosis Promotes Japanese Encephalitis Virus Proliferation in Porcine Kidney 15 Cells

**DOI:** 10.3390/v15040974

**Published:** 2023-04-15

**Authors:** Weimin Xu, Ke Yang, Yi Zheng, Sanjie Cao, Qigui Yan, Xiaobo Huang, Yiping Wen, Qin Zhao, Senyan Du, Yifei Lang, Shan Zhao, Rui Wu

**Affiliations:** Research Center of Swine Disease, College of Veterinary Medicine, Sichuan Agricultural University, Chengdu 611130, China

**Keywords:** JEV, BAK, pyroptosis, GSDMD, inflammatory cytokines

## Abstract

As a zoonotic virus, Japanese Encephalitis virus (JEV) poses a serious threat to human health and the breeding industry. Regarding the mechanism and complications of tissue inflammation caused by JEV, such as encephalitis and orchitis, there is no effective drug treatment currently, and the mechanism of occurrence has not been thoroughly studied. Therefore, it is necessary to study the mechanism of the inflammatory pathway caused by JEV. As one of the key proteins regulating cell death, BCL2 antagonist/killer (BAK) is also a necessary prerequisite for the release of cellular inflammatory factors. We found that after JEV infection, BAK-knockdown cells died less than normal cells, and the transcription levels of inflammatory factors such as *TNF*, *IFNα*, and *IL-1β* and their corresponding regulatory genes were also significantly reduced. By further verifying protein expression on the cell death pathway, it was found that pyroptotic activation and virus titer were also significantly reduced in BAK.KD cells, suggesting that JEV proliferation might be related to BAK-induced cell death. From our data, we could conclude that JEV utilized the BAK-promoted pyroptotic pathway to release more virions after the final Gasdermin D-N (GSDMD-N) protein pore formation for the purpose of JEV proliferation. Therefore, the study of the endogenous cell death activator protein BAK and the final release pathway of JEV, is expected to provide some new theoretical basis for future research on the screening of targeted drugs for the treatment of inflammatory diseases caused by JEV.

## 1. Introduction

Japanese encephalitis virus (JEV) is a single-stranded, enveloped RNA virus of the *Flaviviridae* family. It is mainly found in Asian countries and causes serious brain diseases, especially in children and the elderly [1,2]. JEV is classified into five genotypes based on genetic differences [3]. Bats and birds are the natural hosts of JEV, and pigs and cattle are the amplification hosts of JEV [4,5]. Therefore, the impact of JEV on animal husbandry, especially pig farming, is particularly significant. The currently prevalent strain in Asia is the type III strain, but according to epidemiological surveys, the type I strain has gradually replaced the original strain [4]. With the replacement of new JEV strains, the virulence of their antigens may also change, so it is necessary to further clarify the pathogenic genetic mechanism of the virus to ensure the development of new target gene therapy [6].

In addition to causing animal nervous system diseases, JEV can also cause reproductive disorders in pigs, such as piglet abortion, stillbirth, and fetal mummification [7,8,9]. When the JEV in pigs reaches a certain level, it triggers a storm of inflammatory cytokines in the body, resulting in various tissue inflammatory diseases, such as encephalitis and orchitis [9]. In order to investigate whether there was an inflammatory correlation between virus-induced testicular inflammation and kidney inflammation, and whether the kidney could also induce an inflammatory storm, pig kidney epithelial cells (PK-15) were selected as the research object in this study.

Tissue inflammation caused by viruses is mainly a kind of pathological damage [6]. The virus stimulates cells to produce a large number of cytokines, causing the body’s immune system to overreact, killing normal cells and infected cells [10]. As we know, the BCL2 antagonist/killer (BAK) is one of the key proteins in cell death and has been shown to play a key role in the viral regulation of cell death and inflammatory activation [11]. Taking pyroptosis as an example, when cells are stimulated by death signals, BAK can shed the α2 helix structure and complete activation [12], aggregate in the form of oligomers in the outer mitochondrial membrane to form pores, release second messengers, such as ROS, mtDNA, and cytochrome c; and then activate proteins on subsequent death pathways [13,14]. When released into the cytoplasm, they activate proteins of the cysteinyl aspartate-specific protease (caspase) family [15], which associate with other pro-death proteins, such as ASC and NLRP3, to form inflammasomes [16]. Completely cleavage and activate caspase-1, promote terminal inflammatory factor IL-1β, etc., activate cell pyroptotic porin GSDMD [17,18]. However, regardless of whether it is stimulated by the virus or not, in BAK-mediated downstream death mechanisms, once pyroptosis and apoptosis occur simultaneously, the disease will often be aggravated. When cell apoptosis is dominant and a small part of cell pyroptosis occurs, diseases such as autophagy and a loss of cardiac function will occur, while when cell pyroptosis is dominant, tissue inflammation diseases such as atherosclerosis and sepsis will appear [19,20,21]. Therefore, judging the type of cell death, exploring the mechanism of virus-induced cell death and the purpose of its utilization are great breakthrough points in the current disease treatment.

In this study, CRISPR/Cas9 technology was used to obtain BAK.KD cells, and it was identified that the expression of the BAK protein was significantly reduced. Based on the findings, JEV promoted BAK-mediated MOMP at peak viral titers while activating the Caspase1-NLRP3-GSDMD-mediated pyroptotic pathway and releasing inflammatory cytokines such as IL-1β, IFN, and TNF. In contrast, this pathway was significantly inhibited in BAK.KD cells, and the viral load of JEV was greatly reduced, suggesting that BAK-mediated pyroptosis might be involved in JEV proliferation. This is also the question that this study aims to explore.

In conclusion, JEV also exhibits the ability to modulate inflammatory cytokine pathways when infecting non-neuronal cells. Moreover, when the virus life cycle reaches assembly and release, JEV may induce BAK activation to promote cell pyroptosis and release inflammatory factors while releasing JEV, so as to achieve the purpose of virus proliferation. The final GSDMD protein inhibitor treatment demonstrated a proliferative effect of pyroptosis on JEV proliferation. This may provide a new idea for the targeted therapy of JEV tissue inflammation in the future, and also provide a certain theoretical basis for inhibiting virus death and virus release.

## 2. Materials and Methods

### 2.1. Cells and Virus Infection

The JEV wild-type strain used in this study was isolated from the brain tissues of aborted piglets in 2012, which belongs to genotype I (strain SCYA201201, GenBank: KM658163). Porcine Kidney (PK)-15 cells (ATCC), BAK-Knockdown PK-15 cells (generated from PK-15 cells as parental cells in this study), Human Embryo Kidney (HEK)-293 cells (ATCC) and Baby Hamster Kidney (BHK)-21 cells (ATCC) were maintained in Dulbecco’s Modified Eagle Medium (DMEM; Gibco, Grand Island, NY, USA) supplemented with 10% fetal bovine serum (FBS; Hyclone, South Logan, UT, USA), 100 U/mL penicillin, and 100 µg/mL streptomycin. All cell lines were grown at 37 °C in a 5% CO_2_ humidified atmosphere.

JEV was adsorbed into the monolayers of BHK21 cells at the indicated multiplicity of infection (MOI) for 1 h at 37 °C. An unbound virus was removed by washing with serum-free Dulbecco’s Modified Eagle’s Medium (DMEM). Cells were then overlaid and stored in DMEM containing 2% fetal bovine serum at 37 °C.

### 2.2. Plasmids and Antibodies

The plasmids and antibodies used in this study are listed in Table 1. The cDNA of porcine BAK (NCBI accession no. XM_021098603) was amplified from PK15 cells by PCR. The full-length BAK gene amplified by PCR was inserted into the pcDNA-MYC vector digested with Bam HI and Hind III to construct the pcDNA-MYC-BAK plasmid. The MYC-tagged BAK expression plasmid was used for BAK gene complementation experiments. The sequences of the recombinant plasmids were verified by sanger dideoxy sequencing.

### 2.3. Construction and Characterization of BAK Knockdown PK15 Cell Line by CRISPR/Cas9

The online CRISPR tool (http://chopchop.cbu.uib.no/, accessed on 17 September 2019) used to design the sgRNAs for BAK gene editing sites (sgRNA-F-1: 5′- CACCGCTGGAATTCCGAGTCGTATCGC-3′ and sgRNA-R-1: 5′-AAACGCGATACGACTCGGAATTCCAGC-3′). The two sgRNA dimers were then cloned to the LentiCRISPR-V2 plasmid (Addgene, Watertown, MA, USA). Subsequently, the recombinant plasmids, psPAX (Addgene, Watertown, MA, USA) and pMD2.G (Addgene, Watertown, MA, USA), were co-transfected into HEK-293T cells with Lipofectamine 3000 (Invitrogen, Carlsbad, CA, USA). A control group was set up, and the empty plasmid was transfected in the same way. After 24 h of transfection, the supernatant was collected and filtered to serve as a lentiviral solution carrying the target sgRNA for the infection of PK-15 cells. Finally, puromycin (5.5 μg/mL) was added to select positive cells. Cell lines with BAK knockdown were confirmed by DNA sequencing and Western blotting, and after verifying the difference with the control group cells, monoclonal cell lines were screened for subsequent experiments.

### 2.4. Reactive Oxygen Species Assay

A Reactive Oxygen Species Assay Kit (Beyotime Biotechnology, Shanghai, China) was used to detect cell proliferation in this study. The DCFH-DA probe was loaded into cells and then oxidized by ROS, and the fluorescence signal was captured by the instrument to detect ROS levels. The cells to be tested were placed in a 6-well plate (10^6^ cells/well) and cultured in a 5% CO_2_ incubator at 37 °C, with 3 replicates in each group. Cells were infected with JEV at MOI = 10, and ROS levels were detected at 48 hpi (hours post-infection). A control group was set up in the same way. The DCFH-DA probe was diluted to 10 μmol/L with serum-free DMEM and then added to a 6-well plate (100 μL per well) and incubated for 20 min. Immediately after washing samples with serum-free DMEM, fluorescence signals were detected at an excitation wavelength of 488 nm and an emission wavelength of 525 nm.

### 2.5. Complementation and Overexpression Experiment of BAK Gene

The constructed BAK eukaryotic expression plasmids were used for overexpression and complementation experiments, respectively. In the overexpression experiment, the plasmid was transfected into PK15 cells, and in the complementation experiment, the plasmid was transfected into BAK.KD cells. Three experimental groups were set up: blank group (M, no transfection plasmid into cells), control group (K, transfection of empty vector plasmids into cells), and experimental group (BAK, transfection of plasmid containing BAK gene into cells); the groups were transfected for 24 h. Cells were infected with JEV MOI = 1; after 24 h of post-infection, the cells were collected to detect JEV gene replication by fluorescence quantification.

### 2.6. EDU Cell Proliferation Assay

EDU is a novel thymidine analogue, which can replace thymidine and be incorporated into the newly synthesized DNA. Via the click reaction between the acetylene group on EdU and the fluorescently labeled small molecule azide probe, the newly synthesized DNA is fluorescently labeled, and then cell proliferation can be observed under a fluorescence microscope (BX53, OLYMPUS, Tokyo, Japan). The BeyoClick™ EdU Cell Proliferation Kit with Alexa Fluor 555 (Beyotime Biotechnology, Shanghai, China) was used to detect cell proliferation in this study. All procedures were performed according to the manufacturer’s recommendations.

### 2.7. Detection and Analysis of the Apoptosis and Necrosis of YO-PRO-1/PI Cells

YO-PRO-1 (Oxazole yellow, YP1) is a green fluorescent nucleic acid dye that has no permeability relative to the cell membranes of normal animals, but it can penetrate the cell membranes of apoptotic cells to detect cell apoptosis. It only glows bright green when it binds to DNA. Propidium iodide (PI) is a nucleic acid red fluorescent dye, which can only stain necrotic cells such that they lose their membrane integrity and combine with nucleic acids to produce bright red fluorescence. Therefore, YP1 can be used in combination with PI to detect both apoptotic and necrotic cells. Apoptotic cells show green fluorescence, necrotic cells show both red and green fluorescence positive, and living cells show little or no fluorescence. The YP1/PI cell apoptosis and necrosis detection kit (Beyotime Biotechnology, Shanghai, China) was used in this study, and the final detection was performed by flow cytometry (Flow Cytometry, BDVerse, San Jose, CA, USA). All operations were performed according to the manufacturer’s recommendations.

### 2.8. RNA Extraction and Quantitative Real-Time RT-PCR

The experimental group and control group cells were collected according to the experimental design time. Then, total RNA was extracted using TRI-zol Reagent (Sangon Biotech, Shanghai, China). Reverse transcription was performed using Prime Script™ RT reagent Kit with gDNA Eraser (Perfect Real Time) (TaKaRa Bio, Tokyo, Japan). The resulting cDNA was used for quantitative real-time PCR with specific primers (Table 2) using TB Green^®^ Premix Ex Taq™ II (Tli RNaseH Plus) (TaKaRa Bio) in a Light Cycler^®^ 96 System (Roche, Basel, Switzerland). Relative mRNA values were calculated using the 2^−∆∆CT^ method. GAPDH was used as an internal control in each sample and shown as fold change by normalizing to the mock control.

### 2.9. Western Blot

Cells were washed twice with cold PBS and then incubated for 45 min at 4 °C in a RIPA lysis buffer with PMSF (Solarbio, Beijing, China). Lysates were centrifugated at 12,000 rpm for 5 min at 4 °C, and the supernatants were collected. The supernatant was mixed with a protein loading buffer and boiled at 70 °C for 10 min. After cooling, the BCA concentration of the protein was determined, and the total amount of protein loaded was unified. The treated supernatant was subjected to SDS-PAGE and then transferred to a PVDF membrane (Bio-Rad, Hercules, CA, USA) at 250 mA for 60 min. Membranes were blocked for 2 h at room temperature with 5% (*w/v*) skim milk in TBST (0.05% Tween 20, 0.15 M NaCl, 1 mM Tris-HCI, pH 7.5) and then incubated with antibodies relative to the corresponding proteins overnight. All antibodies were diluted in a primary antibody dilution buffer (Beyotime, Shanghai, China). Membranes were washed four times with TBST for 4 min each and incubated with 1:5000 HRP-conjugated goat anti-rabbit IgG for 45 min at room temperature. Membranes were washed again, and bands were developed by adding ECL (Bio-Rad, Hercules, CA, USA) according to the manufacturer’s instructions.

### 2.10. Indirect Immunofluorescence (IF) Assay for the Detection of GSDMD and JEV E

Cells were seeded on coverslips, and when the cells grew to 90%, they were infected with JEV at MOI = 1, and a blank control group was set up. Then, 48 h after infection, the cells were fixed with 4% paraformaldehyde, and the cells were permeabilized and sealed by conventional methods after fixation. Anti-GSDMD (ABclonal, Wuhan, China) and anti-JEV E (homemade) primary antibodies (1:200) were then incubated overnight at 4 °C. After washing, cells were incubated with FITC-conjugated and CY3-conjugated secondary antibodies (1:500) (ABclonal) for 1 h at room temperature. Finally, nuclei were stained with Hoechst and visualized with a fluorescence microscope.

### 2.11. Pyroptosis Inhibitor Disulfiram

Disulfiram, also named tetraethylthiuram disulfide, has recently been found to specifically bind to and inhibit the formation of the pore structure formed by GSDMD proteins, so it was used in the inhibition experiment of cell pyroptosis [22,23]. Disulfiram (selleck, NSC 190940, catalog number: S1680, alias: NSC 190940, Tetraethylthiuram disulfide, TETD) was dissolved in DMSO to make a 10 mM stock solution and then diluted with DMEM to 0 μM, 10 μM, 25 μM, 50 μM, and 75μM; these five concentrations were added to PK15 cells. After a 48h wait, cell death was measured with CCK8. Finally, the optimal working concentration of disulfiram was selected.

### 2.12. Quantification and Statistical Analysis

All statistical analyses and calculations were performed using GraphPad Prism (Software Version 8, San Diego, CA, USA). Three independent replicates were set for each experiment, and the experimental results were averaged for processing. All data are expressed as means ± standard deviation (SD) as indicated. A Student’s unpaired t test was used to estimate the statistical significance between two groups, whereas ANOVA was used to compare the means among three or more groups. A *p*-value of less than 0.05 was considered statistically significant. Significant differences between groups are indicated by * *p* < 0.05, ** *p* < 0.01, *** *p* < 0.001, and **** *p* < 0.0001.

## 3. Results

### 3.1. Proliferation of JEV Is Inhibited in BAK.KD Cells

**Figure 1:**
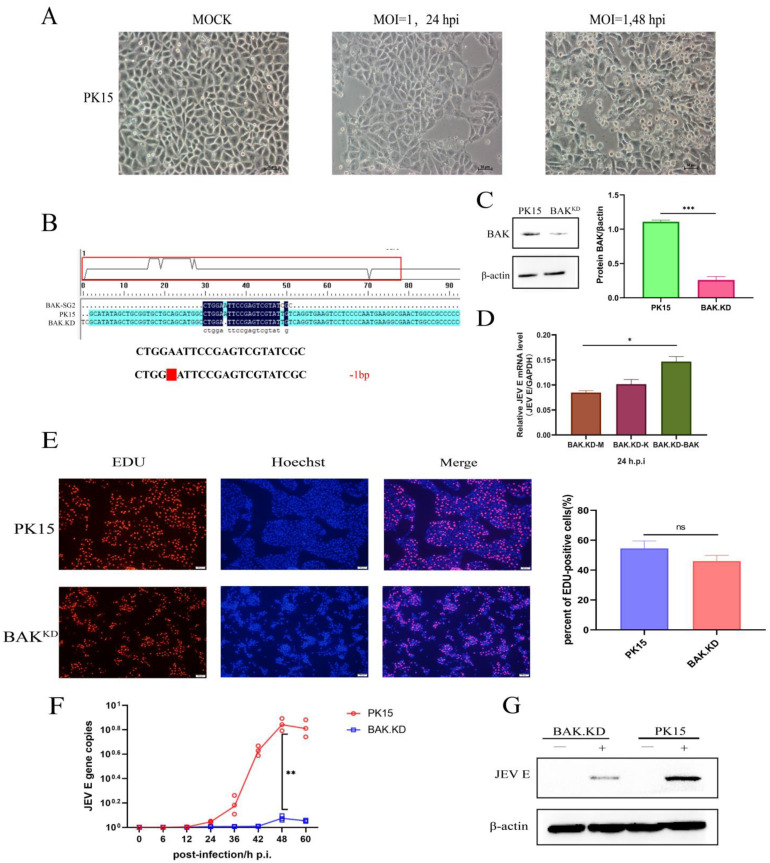
Proliferation of JEV is inhibited in BAK.KD cells. (**A**) Normal PK15 cells were infected with JEV at MOI = 1, and cell death was observed at 24 and 48 h. (**B**) Sequence alignment between BAK.KD cells and normal cells (located at the porcine-derived BAK whole-genome DNA (No.29811711–No.29811736). A red blank represents a missing base. (**C**) WB identification of BAK protein expression in BAK.KD and normal cells; knockdown resulted in extremely significant protein reduction *** (*p* < 0.001) using grayscale quantification. (**D**) The BAK eukaryotic expression plasmid was transfected into cells for BAK gene complementation experiments. M, blank group; K, empty vector plasmid group; BAK, experimental group transfected with BAK plasmid. (**E**) PK-15 cells and BAK.KD were loaded with EdU and incubated for 2 h. After waiting for the reaction, photographs were taken with a fluorescent microscope, EdU-positive cells are colored in red, and nuclei are colored in blue. At least 3 photos were taken of each sample. Finally, the percentages of EdU-positive cells were calculated separately, and the average was taken as the final data. (**F**) Cells in a T75 cell flask were infected with JEV virus titers at MOI = 1. After replacement with 20 mL of 2% serum DMEM as the maintenance solution, 500 µL of supernatants was collected at different times, and viral RNA was extracted and reverse transcribed into cDNA. Viral copy number changes were detected by absolute fluorescence quantification, and growth curves were drawn. (**G**) Western blot was used to quantify proteins in cells. The experimental (JEV, MOI = 1, 48 hpi) group and control group illustrate the expression level of JEV E protein. All samples were run in triplicate, and the results were averaged for processing. * (*p* < 0.05), ** (*p* < 0.01), *** (*p* < 0.001), and ns (not significant).

When PK15 cells were infected with JEV, we chose different time points to observe the changes in cell states and confirmed the effect of JEV on cell death (Figure 1A). Then, the genes of infected cells were analyzed by transcriptome sequencing, and we found that the impact score of *BAK* gene transcription was higher in JEV-infected cells. To further explore the mechanism of influence, we chose to knockdown BAK. Using the online CRISPR editing website (http://crispr.mit.edu/, accessed on 17 September 2019), a pair of sgRNAs targeting the porcine *BAK* gene were designed, and the BAK.KD cell line with one base missing in the original sequence was obtained (Figure 1B). BAK.KD cells were tested and validated (Figure 1C), demonstrating a significantly reduced level of BAK protein expression (*p* < 0.001). Although there might still be a small amount of BAK protein, a significant reduction in the level of JEV gene replication had been found in BAK.KD cells. We also constructed a BAK eukaryotic expression plasmid and exogenously induced BAK expression. During BAK gene complementation, JEV gene duplication was found to increase with BAK (Figure 1D). PK-15 and BAK.KD cells were then incubated with EDU for 2 h under the same conditions. No significant differences were found in the percentage of EDU-positive cells under fluorescence microscopy, suggesting that BAK.KD did not have differential effects on cell proliferation (Figure 1E). Subsequently, BAK.KD and normal cells were infected with JEV virus titers at MOI = 1, and the growth curves of JEV were measured. The results are shown in Figure 1F, the viral copy number of JEV in normal cells peaked at 48 h, and the proliferation of JEV was significantly inhibited in BAK.KD. Meanwhile, at the protein level (MOI = 1, 48 hpi), it was also shown (Figure 1G) that the JEV E protein was significantly inhibited. It was observed that the BAK could affect the proliferation of JEV.

### 3.2. BAK-Mediated Cytokine Transcription Levels Significantly Increased in the Late Stage of JEV Proliferation

**Figure 2:**
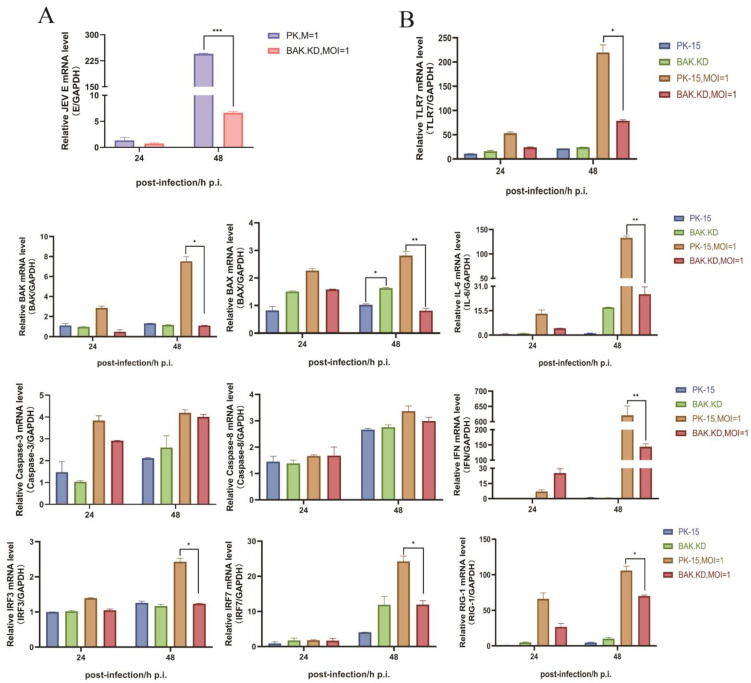
BAK-mediated cytokine transcription levels were significantly increased in the late stage of JEV proliferation. (**A**) JEV-infected normal cells and BAK.KD cells were collected, the total RNA was extracted and reverse transcribed into cDNA, and the expression of the intracellular E gene was measured at 24 and 48 h. (**B**) Normal cells and BAK.KD cells were also quantitatively determined by the fluorescence quantitative determination of JEV-induced changes in the transcriptional levels of inflammatory cytokine genes, with *GAPDH* as the reference gene. Three independent replicates were set for each experiment, and the experimental results were averaged for processing. * (*p* < 0.05), ** (*p* < 0.01), *** (*p* < 0.001), and ns (not significant).

According to virus genome copy numbers, we chose 24 h and 48 h as the time points for detecting gene changes in order to detect changes in the transcriptional levels of BAK-related cytokine genes after JEV infection. We detected the relative content of JEV E genes in infected cells (Figure 2A) and found that the proliferation rate of JEV in BAK.KD was much lower than that of normal cells, which was consistent with the trend of the virus’s growth curve. The results of inflammatory gene detection are shown in Figure 2B. During the 24 h period of JEV infection, the inflammatory factors in the cells show an upward trend. However, the transcription levels of cytokine genes measured at 48 h changed significantly, including caspase family proteins, interleukin family, interferon and other pathway proteins, indicating that the JEV infection induced cellular autoimmune responses, resulting in a large number of inflammatory responses. After infection with JEV, the gene transcription levels of *caspase-3* and *caspase-8* showed an upward trend in both normal cells and BAK.KD cells, but whether this is related to JEV-induced apoptosis needs further verification. In addition, under the stimulation of JEV infection, the transcription level of *BAK* gene in normal cells significantly increased, but after the knockdown of the *BAK* gene, the transcription level of the JEV E gene significantly decreased. It was speculated that BAK might promote the proliferation of JEV. Furthermore, we found that a reduced *BAK* expression might lead to increased *BAX* expression to complement BAK function and activate subsequent pathways. Although the transcription of other related inflammatory cytokines increased, it was still far below the normal cellular level and was significantly suppressed. It was also shown that in the late stage of JEV proliferation, it promoted cell death by inducing BAK (mainly BAK protein).

### 3.3. Knockdown of BAK Function Inhibits JEV-Activated ROS and Other Cytokines

**Figure 3:**
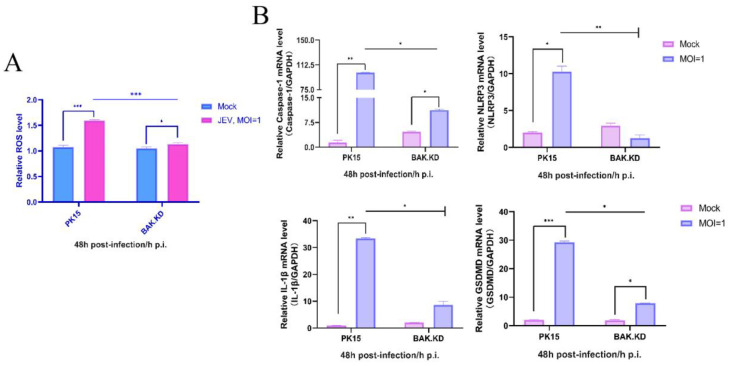
Knockdown of BAK function inhibits JEV-activated ROS and other cytokines. (**A**) DCFH-DA probes were loaded into normal and knockdown cells infected with JEV (MOI = 1, 48 hpi, experimental group) and controls. Fluorescence signals were collected at an excitation wavelength of 488 nm and emission wavelength of 525 nm, relative levels of intracellular ROS were calculated, and data were normalized and displayed as fold change (the data of the control group and the experimental group were normalized at the same time). (**B**) The relative mRNA levels of *Caspase-1, NLRP3, IL-1β,* and *GSDMD* in BAK.KD after JEV infection (MOI = 1, 48 hpi) were detected by real-time quantitative RT-PCR, and PK-15 cells were used as the control. * (*p* < 0.05), ** (*p* < 0.01), *** (*p* < 0.001), and ns (not significant).

Based on changes in inflammatory genes, we suspected that JEV might induce pyroptosis. Therefore, we chose to measure changes in pyroptotic genes and ROS at 48 h from the peak of JEV proliferation. According to the ROS assay results (Figure 3A), we found that under normal conditions, there was little difference in the amount of ROS between PK15 and BAK.KD cells. However, after JEV infection, the amount of ROS released in normal cells was significantly higher than that of those in BAK.KD cells, indicating that JEV did induce the MOMP. Then, gene changes in ROS-related subsequent inflammatory pathways were measured by relative fluorescence quantification (Figure 3B), and the results showed that the transcription levels of genes in the ROS-activated Caspase1-NLRP3-GSDMD pathway significantly increased. We speculated that the JEV infection did induce BAK-mediated upstream MOMP and downstream NLRP3-mediated pyroptosis in PK15 cells.

### 3.4. The Activation of NLRP3 Inflammatory Pathway Proteins Induced by JEV Is Significantly Inhibited in BAK.KD Cells

**Figure 4:**
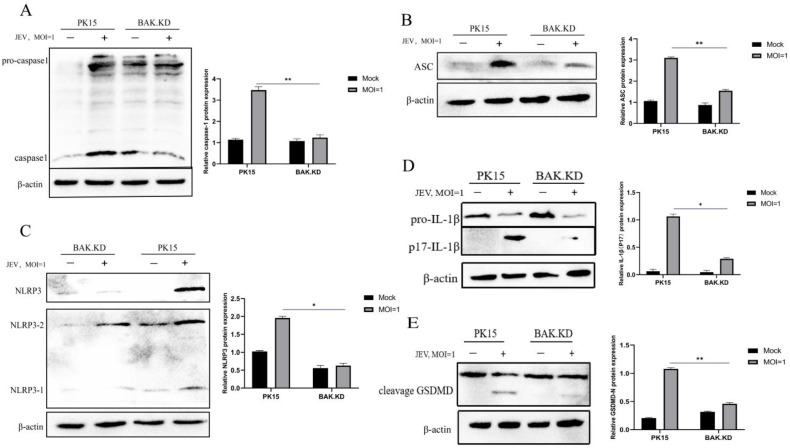
The activation of NLRP3 inflammatory pathway proteins induced by JEV is significantly inhibited in BAK.KD cells. Changes in protein levels during late pyroptosis were determined by Western blotting. The experimental group (JEV, MOI = 1, 48 hpi) and the control group (Mock) exhibited the expression levels of several endogenous proteins. (**A**) Caspase-1, (**B**) ASC, (**C**) NLRP3, (**D**) IL-1β, (**E**) GSDMD, and β-actin as an internal control. Three independent replicates were set for each experiment, and the experimental results were averaged for processing. * (*p* < 0.05), ** (*p* < 0.01).

We identified changes in the transcriptional levels of genes affecting the pyroptotic pathway following JEV infection, and we measured changes in protein levels next (cleavage and activation of proteins). According to the different degrees of the protein display of JEV (MOI = 1, 48 hpi) measured by WB (Figure 4A–E), results for each protein were analyzed separately by gray scale quantification, and a significant differential cleavage of Caspase-1, NLRP3, ASC, and IL-1β compared to the normal group was found, and these were inhibited in BAK.KD. The inhibition of this cleavage was also evident in the release channel formed by the final GSDMD protein. This suggested that the Caspase1-NLRP3-GSDMD pathway was activated to release inflammatory cytokines in the later stages of the JEV life cycle, which might also be a key cause of tissue inflammation. Moreover, the activation of this inflammatory response could accelerate cell rupture and death, which might be more conducive to the release and proliferation of viruses.

### 3.5. Activation of the Pyroptotic Pathway May Promote the Release of JEV

**Figure 5:**
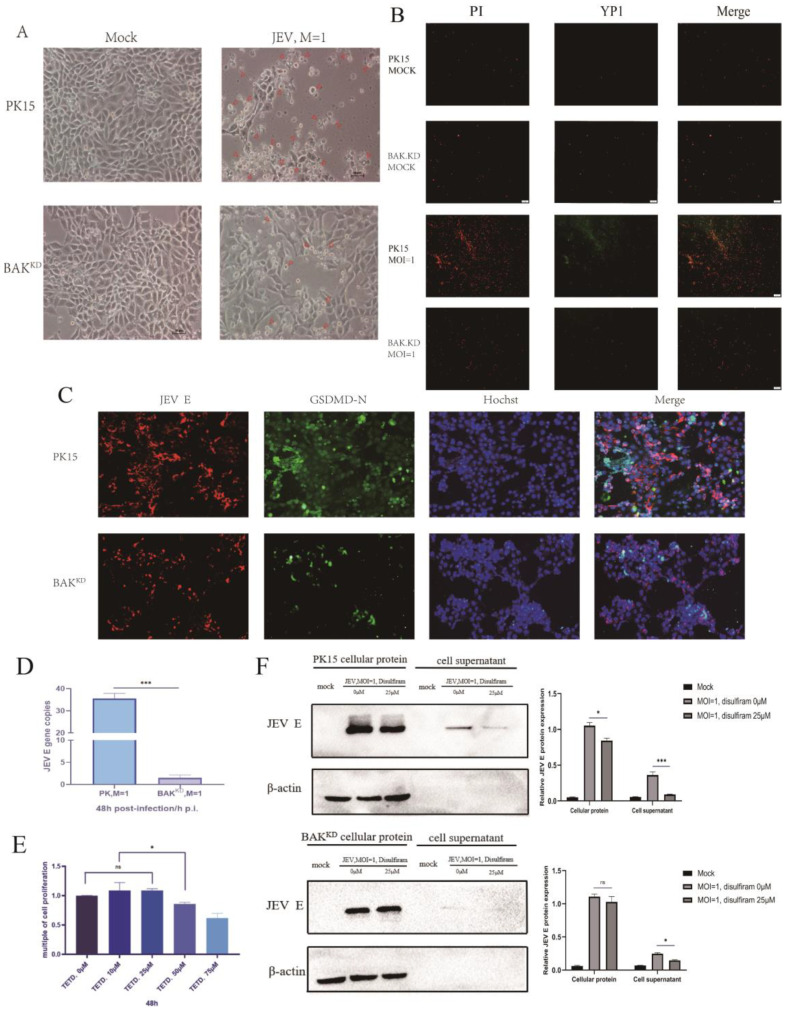
Activation of the pyroptotic pathway may promote the release of JEV. In this study, (**A**) 48 h after the JEV infection of cells, state changes in the cells were observed. The small arrows marked swollen cells, the damaged cell membrane, and the formation of intracellular bubbles. (**B**) The 12-well plate was plated with cell slides, and then the cells were added. When the cells reached 90%, the cells were infected with JEV MOI = 1 for 48 h. Afterwards, the cells were treated according to the fluorescence quantitative microscope observation method of the PI/YP1 apoptosis and necrosis detection kit and then observed under a fluorescence microscope. Red fluorescence indicated necrotic and apoptotic cells with damaged cell membranes, and green fluorescence indicated apoptotic cells with unbroken cell membranes. (**C**) Cells were incubated with a mouse anti-JEV E protein and rabbit anti-GSDMD-N at a dilution of 1:300 for 2 h, washed 3 times with PBST, and then incubated again with FITC-Goat-Anti-Rabbit and Cy3-Goat-Anti-Mouse; the cells were then diluted 1:500 for 1 h, washed in the same way, and the nuclei were stained with Hoechst for 10 min. Finally, the colors were observed with a fluorescence microscope. (**D**) The supernatant of PK15 and BAK.KD cells infected with JEV for 48 h was collected, the viral RNA in the supernatant was extracted and reverse transcribed, and the fold ratio of JEV E in the supernatant was determined by absolute fluorescence quantification. (**E**) Different concentrations of the inhibitor disulfiram were pre-incubated with normal cells and exhausted cells for 48 h to determine the minimum concentration and time at which cells started to die. (**F**) The cells were infected with JEV MOI = 1, and 25 μM disulfiram was added for 48 h. Afterwards, the cell supernatant and cell protein were collected, and the change in the JEV E protein content was verified by WB. The changes in the viral E protein content of the two cells were independently determined by greyscale quantification. Three independent repetitions were set up for each experiment, and the verification results were consistent with the experimental results. * (*p* < 0.05), *** (*p* < 0.001), and ns (not significant).

At the protein level, it was demonstrated that the JEV infection induced an increase in the expression of proteins in the pyroptotic pathway. In addition, by observing the pathological morphology of cells (Figure 5A), JEV-infected cells showed obvious cell swelling, forming bubble-like protrusion structures, and a loss of cell membrane integrity, speculating that JEV mainly induced cell necrosis and cell pyroptosis. Cell death was observed by fluorescence microscopy via PI and YP1 staining (Figure 5B), green fluorescence indicated the number of apoptotic cells, and red fluorescence indicated the number of necrotic and pyroptotic cells, indicating that JEV-induced apoptosis accounted for a small part but mainly pyroptosis and necrosis. Although BAK.KD also showed increased death after infection with JEV, the trend of increase was not large, and this death might be caused by the regulation of other cell death pathways. Since both necrosis and pyroptosis belong to membrane damage, it was temporarily impossible to distinguish them. Therefore, we observed the localization relationship between pyroptotic proteins and JEV by indirect immunofluorescence (Figure 5C). Compared with normal cells, the levels of activated GSDMD proteins and viruses were lower in BAK.KD cells, suggesting that the reduced expression of BAK inhibited pyroptosis and virus proliferation. In normal cells, JEV infection not only showed abundant GSDMD protein activation but also fluorescence overlapping with JEV, suggesting that JEV has a certain promoting effect on this pyroptosis mode. To determine the titer of viruses released by cells, JEV-infected normal cells and BAK.KD supernatants were collected for 48 h to determine viral gene copy numbers (Figure 5D). The results confirmed our conjecture that the virus in the BAK.KD supernatant was significantly reduced, indicating that the release of JEV was inhibited.

Finally, to demonstrate the effect of JEV on pore formation of the GSDMD-N protein during pyroptosis, we intended to use the GSDMD pore formation inhibitor disulfiram (Selleck, catalog number: S1680, tetraethyl thiuram disulfide) to verify the conjecture. After treating normal cells and BAK.KD with different concentrations of disulfiram, we collected supernatants and cells, and we found that the JEV release decreased with increasing concentrations of the pyroptosis inhibitor disulfiram (Figure 5E), indicating that the release of JEV utilized the pores formed by GSDMD. However, JEV was still detected in the supernatant after using the inhibitor. We speculated that the members and functions of the GSDM protein family might not be completely inhibited, such as GSDME, GSDMB, etc., or the cells might rupture via other methods of death, so the release of JEV could not be completely inhibited.

## 4. Discussion

Since its discovery, the infection hosts of JEV include humans and large livestock, and the pathological diseases are diverse, which brings a huge burden to social health and agricultural development, so it has received extensive attention [24]. Therefore, it is necessary to explore the molecular mechanism of JEV-induced cell inflammation and the impact of JEV on inflammation in other tissues.

**Figure 6:**
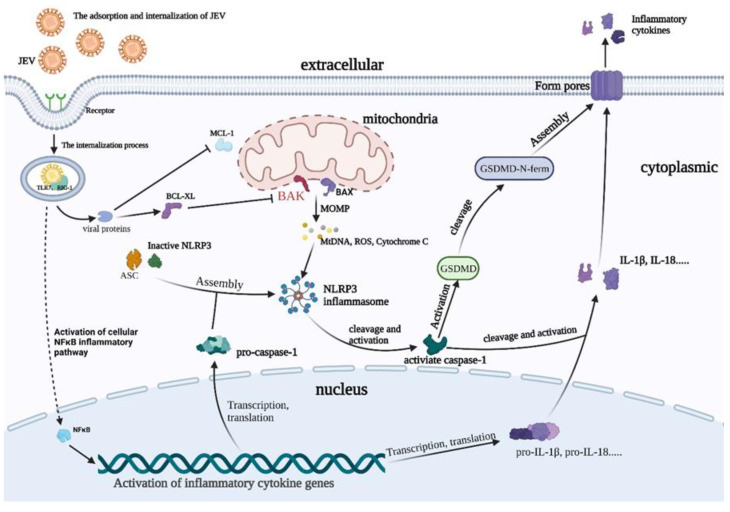
When JEV enters the release phase of the life cycle, JEV can promote BAK activation to activate the MOMP process. Subsequently, the release of ROS promotes the combination of pro-caspase-1, ASC, and pro-NLRP3 to form the NLRP3 inflammasome, forming an activated form of caspase-1. Then, caspase-1 activates inflammatory factors such as IL-1β and cleaves the N-segment of the GSDMD protein. Finally, the GSDMD-N protein aggregates on the cell membrane to form pores, releasing IL-1β and virions.

During infection, a large number of cells rupture and die, and the release of inflammatory factors has been identified as one of the important causes of tissue inflammation [25]. BAK-regulated MOMP is a key point that ultimately leads to cell death, including apoptosis and pyroptosis (Figure 6). This regulation plays an important role in tumor development and neurodegenerative and autoimmune diseases [26]. When BAK is activated by the apoptotic signal, it will aggregate into oligomers, form pores on the mitochondrial outer membrane, and then release ROS and cytochrome C [27,28]. Once these factors enter the cytoplasm, they activate the caspase family of proteins, cascading the activation of the final cell death pathway. The activation of BAK is strongly associated with the final cell death pathway, especially after viral infection [29]. It has been found that a variety of viruses infect cells, and BAK can be used to regulate the time and scale of cell death, thereby achieving virus proliferation [30]. While this may be just the tip of the iceberg of cellular utilization by viruses, it also highlights the important role of BAK in influencing the life cycle of certain viruses. Our results showed that the activation of BAK in normal cells promoted the release of cellular inflammatory factors, while the viral load and inflammatory cytokines in BAK.KD cells significantly decreased, indicating that BAK promoted the release of inflammatory cytokines. In other words, pyroptosis might promote JEV proliferation.

In this study, we successfully constructed a BAK.KD cell line. The fluorescence quantification of gene changes in JEV-infected normal and BAK.KD cells was determined in different time periods. These data showed that JEV could stimulate the activation of NFκB upstream genes *IRF3* and *IRF7*, and the upstream genes of interferon pathway *RIG-1* and *TLR7*; the caspase family, such as *caspase-1* and *caspase-9*; and other genes significantly increased after infection in normal cells, indicating that the inflammatory responses of cells did become stimulated after JEV infection. However, after the JEV infection of BAK.KD cells, these genes were significantly repressed to varying degrees. Considering that the presence of BAX after BAK knockdown may complement the pro-apoptotic function of BAK, as well as the activation of other inflammatory pathways, it is reasonable to explain the reason for the upward trend of inflammatory factors in BAK.KD cells [27,31]. By measuring the gene expression of BAK and BAX, we found that the amount of the BAK gene was lower in BAK.KD cells, while the expression of BAX changed little in normal cells and increased significantly in BAK.KD cells, confirming our suspicion. Meanwhile, by exogenously expressing BAK and BAX, we found that BAX did not have much difference in JEV gene replication, so we paid more attention to the induction of BAK.

When examining genetic changes in inflammatory factors in infected cells, we also found changes in BAK-mediated cell death pathway genes. After BAK forms pores and disrupts the mitochondrial outer membrane, ROS and cytochrome c are released, which is key to activating the pyroptotic process [32,33]. ASC is then activated by ROS to form four substructures. The substructures are combined with pro-caspase1 and the NLRP3 precursor protein to form inflammasome NLRP3, which cleaves and activates caspase-1. The production of IL-1β and GSDMD-N to form pores to release inflammatory factors is further activated [17,34]. We measured protein level changes on the pyroptotic pathway by WB and found that these proteins were clearly cleaved and activated. In addition, the content of inflammatory factors such as IL-1β also increased significantly, indicating that JEV had an inflammatory stimulating effect on PK15 cells, but this effect was significantly weakened in BAK.KD cells, indicating that the inflammatory activation response of BAK relative to JEV played an important role in PK15 cells. In order to explore the release process of the virus, indirect immunofluorescence was used to find the co-localization of JEV protein and GSDMD-N, indicating that there was a certain connection between GSDMD-N pores and JEV release. The virus was significantly reduced in BAK.KD cells by collecting the supernatant and measuring the virus’s content, indicating that JEV may rely on the GSDMD-N pathway to release viruses but not completely. To prove our hypothesis, we used an inhibitor (disulfiram) to inhibit the pore formation of the GSDMD-N protein and measured the virus content in the cell supernatant to estimate whether the release of the virus was inhibited. We chose to treat JEV-infected cells with a disulfiram concentration of 25 μM (the concentration with the highest inhibitory efficiency) and collected supernatants and cell samples for 48 h. We demonstrated by WB that the release of the virus was indeed significantly reduced after the use of the inhibitor, while the virus content in the cells was not much different. It showed that JEV did use this pore to release virus particles while inducing pyroptosis. In this way, cell death led to the release of more JEV and inflammatory factors, which were more likely to cause peripheral inflammatory responses [22,23,35]. Although an inhibitor was used, there was still a way to release JEV. We speculated that the inhibitory effect of disulfiram might only be on the pore formed by the GSDMD-N protein and had little inhibitory effects on other members of the GSDM family. All the above results indicated that JEV utilizes the effect of cell death to realize self-proliferation. We only proved that JEV promoted pyroptosis; whether there is an interaction between the JEV protein and other GSDM family members [36] and the mechanism and type of the interaction are what we need to further explore in the future.

In conclusion, this study demonstrated that JEV could infect PK15 cells and promote the release of inflammatory factors. During the JEV assembly stage, the BAK-mediated NLRP3 inflammatory pathway is activated to promote cell pyroptosis and release JEV to achieve proliferation. BAK is an important protein that JEV utilizes and regulates in intracellular cell death, which can provide some ideas for the development of targeted drugs that inhibit tissue inflammation caused by JEV.

## Figures and Tables

**Table 1:** Plasmids and antibodies.

Plasmid and Antibody	Source	Identifier
pCDNA-MYC-BAK	Research Center of Swine Disease	N/A
FITC Goat Anti-rabbit IgG	ABclonal Technology	Cat#AS011
Cy3 Goat Anti-Mouse IgG	ABclonal Technology	Cat#AS008
Anti-BAK Rabbit polyclonal antibody	ABclonal Technology	Cat#A0498
Anti-ASC Rabbit polyclonal antibody	ABclonal Technology	Cat#A11433
Anti-cleaved N-terminal GSDMD antibody	Abcam	Cat#Ab215203
Anti-NLRP3 Rabbit polyclonal antibody	ABclonal Technology	Cat#A5652
Anti-IL-1β Rabbit polyclonal antibody	ABclonal Technology	Cat#A11369
Anti-Caspase-1 Rabbit polyclonal antibody	ABclonal Technology	Cat#A0964
Anti-β-actin Rabbit polyclonal antibody	ABclonal Technology	Cat#AC038
Anti-JEV E Mouse polyclonal antibodies	Research Center of Swine Disease	N/A

**Table 2:** Primers used for qRT-PCR.

Gene	Sequence (5′-3′)	Length (bp)
JEV E	F: GGGAAGGGAAGCATTGAC R: AAGGAGCATTGGGTGTTA	231 bp
BAK	F: TCAACCGGCGATACGACT R: GTAGCCAAAGCCCAGAAGAG	155 bp
BAX	F: GCAGCCGATCTCGAAGGA R: GCCGAAATGTTTGCTGACG	154 bp
IRF3	F: AAGAAGCATTGCGTTTAGC R: AGGTACTGTATCTGCCATGAG	138 bp
IRF7	F: CATGGGGCGTGGATCTGA R: GCACCGTTCGACCTTTGT	234 bp
TLR7	F: TAACCTCAGTCAACCGCAAGT R: CCATCTTTGGGGCACATAC	190 bp
TRANS	F: AGACGACTGCTGCCATGGA R: ACACCCTGCTGTTTTTCTACCT	155 bp
RIG-1	F: TCCCAGCAACGAGAACCCT R: TTCGTTTTGCCACGTCCAG	195 bp
TNF	F: GGCATTGGCATACCCACTCT R: GCCCAAGGACTCAGATCATC	178 bp
IFNα	F: GGACTCCATCCTGGCTGTGA R: GACTTCTGCCCTGATGATCTCC	103 bp
IL-1β	F: ATGTGGACCTCTGGGTATGG R: ACAAAAGCCCGTCTTCCTG	188 bp
IL-6	F: GGCATCACCTTTGGCATCTT R: CTGCTTCTGGTGATGGTACTG	204 bp
GSDMD	F: ACATGGCCTCCAAGGTAAGA R: GATCGAGTTGGGGCTGTGACT	120 bp
NLRP3	F: AATGGATGGGTTTACTGG R: GGTGAAGCGTTTGTTGAG	189 bp
Caspase-1	F: GTCCGAAGCGGTGAGATT R: CCCAGACATAGCCCAAAG	220 bp
Caspase-3	F: ACCCGAGTAAGAATGTGC R: GACTGTGGGATTGAGACG	212 bp
Caspase-8	F: CTCCTCCTCATTGGTTTCC R: TCCTGAGCCTGGACTACAT	196 bp
GAPDH	F: ACATGGCCTCCAAGGTAAGA R: GATCGAGTTGGGGCTGTGACT	202 bp

## Data Availability

The datasets supporting the conclusions of this study are included in the article.
